# Traffic, Susceptibility, and Childhood Asthma

**DOI:** 10.1289/ehp.8594

**Published:** 2006-02-16

**Authors:** Rob McConnell, Kiros Berhane, Ling Yao, Michael Jerrett, Fred Lurmann, Frank Gilliland, Nino Künzli, Jim Gauderman, Ed Avol, Duncan Thomas, John Peters

**Affiliations:** 1 Department of Preventive Medicine, Keck School of Medicine, University of Southern California, Los Angeles, California, USA; 2 Sonoma Technology Inc., Petaluma, California, USA

**Keywords:** air pollution, asthma, child, epidemiology, traffic

## Abstract

Results from studies of traffic and childhood asthma have been inconsistent, but there has been little systematic evaluation of susceptible subgroups. In this study, we examined the relationship of local traffic-related exposure and asthma and wheeze in southern California school children (5–7 years of age). Lifetime history of doctor-diagnosed asthma and prevalent asthma and wheeze were evaluated by questionnaire. Parental history of asthma and child’s history of allergic symptoms, sex, and early-life exposure (residence at the same home since 2 years of age) were examined as susceptibility factors. Residential exposure was assessed by proximity to a major road and by modeling exposure to local traffic-related pollutants. Residence within 75 m of a major road was associated with an increased risk of lifetime asthma [odds ratio (OR) = 1.29; 95% confidence interval (CI), 1.01–1.86], prevalent asthma (OR = 1.50; 95% CI, 1.16–1.95), and wheeze (OR = 1.40; 95% CI, 1.09–1.78). Susceptibility increased in long-term residents with no parental history of asthma for lifetime asthma (OR = 1.85; 95% CI, 1.11–3.09), prevalent asthma (OR = 2.46; 95% CI, 0.48–4.09), and recent wheeze (OR = 2.74; 95% CI, 1.71–4.39). The higher risk of asthma near a major road decreased to background rates at 150–200 m from the road. In children with a parental history of asthma and in children moving to the residence after 2 years of age, there was no increased risk associated with exposure. Effect of residential proximity to roadways was also larger in girls. A similar pattern of effects was observed with traffic-modeled exposure. These results indicate that residence near a major road is associated with asthma. The reason for larger effects in those with no parental history of asthma merits further investigation.

Childhood asthma prevalence and incidence have been associated with local variation in traffic patterns within communities in many (Brauer et al. 2002; Gauderman et al. 2005; Nicolai et al. 2003; van Vliet et al. 1997; Venn et al. 2000; Zmirou et al. 2004) but not all (English et al. 1999; Waldron et al. 1995; Wjst et al. 1993) studies that have examined the impact of local traffic or traffic-related air pollutants near children’s homes. However, many studies did not evaluate exposure at early age, which may be an important determinant of risk from traffic-related pollution (Zmirou et al. 2004) and which might vary depending on residential stability of study participants. The duration of residence at the same home might also be expected to increase any risk of asthma associated with traffic-related exposure. Other characteristics that might make children more susceptible to this exposure include parental history of asthma and childhood allergy, which are strong risk factors for asthma (London et al. 2001; Peden 2000). A recent study found larger associations of traffic with asthma in children without a parent with asthma (Gordian et al. 2005), and we have previously found that children with incident asthma associated with ozone were less likely to have a parental history of asthma than were other children with asthma (McConnell et al. 2002). Susceptibility to second-hand tobacco smoke exposure, another environmental combustion product, and traffic-related pollutants has been found to vary by atopy in some studies that have examined this relationship (Janssen et al. 2003; Kershaw 1987; Palmieri et al. 1990; Strachan and Cook 1998; Strachan et al. 1996a, 1996b; Zmirou et al. 2004). Some evidence also suggests that girls may be more susceptible than boys to traffic-related exposure (Oosterlee et al. 1996; Pershagen et al. 1995; Shima et al. 2003; van Vliet et al. 1997; Venn et al. 2001).

Concentrations of pollutants in fresh vehicular exhaust are high near roadways but decline markedly within 150–300 m (Gilbert et al. 2005; Zhu et al. 2002). Accurate assessment of this large but very local variation in exposure may be important to identify health hazards. One promising approach has been to estimate residential distance to a major roadway. This can be done with relatively little error in measurement, using geographic information systems and accurately located roadways. Some studies have found increased asthma prevalence in children living within 100 m of a major road, and there is evidence that the risk increases dramatically within 75 m (van Vliet et al. 1997; Venn et al. 2001).

In this population-based study, we examined characteristics that might increase childhood susceptibility to the effects of traffic-related air pollution in a new cohort in the southern California Children’s Health Study, an ongoing longitudinal evaluation of air pollution and respiratory health (Künzli et al. 2003). We evaluated whether parental history of asthma and child age at exposure, symptoms of allergy, and sex influenced susceptibility to the risk of childhood asthma and wheeze associated with exposure to traffic.

## Materials and Methods

### Population.

**Figure 1:**
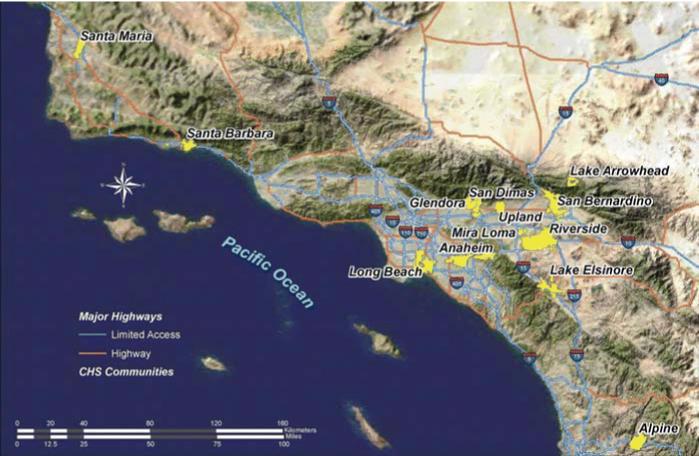
Location of study communities.

A new cohort was recruited in 2003 from schools in 13 southern California communities (shown in Figure 1). Nine communities were the same as in the original Children’s Health Study cohorts, and four were new. All students present in 2003 in all kindergarten and first grade classrooms (5–7 years of age) in participating schools were given a questionnaire and informed consent to take home for parents to complete. Informed consent, approved for this study by the University of Southern California Institutional Review Board, was obtained, and questionnaires were completed and returned for 5,341 (65%) of 8,193 eligible children.

### Assessment of exposure to traffic-related pollutants.

We estimated distance of each participant’s residence to the nearest major road, including freeways, other highways, and arterial roads. Participant residence addresses were standardized, and their locations were geocoded to 13 m perpendicular to the side of the adjacent road, using the Tele Atlas Multinet road network data (Tele Atlas Inc., Menlo Park, CA). Distance to the nearest major road was estimated using ArcGIS software (version 8.3; Environmental Systems Research Institute Inc., Redlands, CA). Each direction of travel was represented as a separate roadway, and the shortest distance was estimated from the residence to the middle of the nearest side of the freeway or major road. We included in the analysis only children with addresses that could be accurately geocoded. Specifically, only residential addresses for which the Tele Atlas geocoding software assigned its highest-quality match code were included. These addresses are located on the correct side of the street with their relative position between cross-streets determined by linear interpolation of residence number between the nearest intersections.

Residential distance to a major road was categorized as < 75 m, 75–150 m, > 150–300 m, and > 300 m, based on results of previous studies showing markedly increased exposure and risk of asthma within 75 m of large roadways, which decreased to background levels by 150–300 m (Gilbert et al. 2005; van Vliet et al. 1997; Venn et al. 2001; Zhu et al. 2002). We also estimated residential exposure to fresh traffic-modeled pollutants from local freeway and nonfreeway sources, accounting for traffic volume, wind speed, and direction in each community, using a line source dispersion model, as described in the accompanying online supplemental material (http://www.ehponline.org/members/2006/8594/suppl.pdf).

### Health outcomes and other questionnaire information.

We classified lifetime asthma based on a questionnaire response to the question “Has a doctor ever diagnosed this child as having asthma?” Current wheeze was defined to include children with any wheezing in the previous 12 months [International Study of Asthma and Allergies in Children (ISAAC) Steering Committee 1998]. Prevalent asthma was defined as the reported use of controller medications for asthma (inhaled corticosteroids, leukotriene inhibitors, cromolyn sodium, or long-acting beta agonists) in the previous year or lifetime asthma with any wheeze in the previous year. In addition, children without a physician’s diagnosis who had severe wheeze in the previous 12 months were included as prevalent asthmatics to identify asthma undiagnosed because of poor access to medical care. Severe wheeze included four or more attacks of wheeze, one or more nights per week of wheeze, or wheeze with shortness of breath so severe as to interfere with speech (ISAAC Steering Committee 1998).

We collected personal and family covariates and housing characteristics by questionnaire, including child’s race and date of birth and the language in which the questionnaire was completed (Spanish or English). Potentially susceptible groups were identified based on child’s sex, allergic characteristics defined as a history of hay fever or a problem with sneezing or runny or blocked nose when the child did not have a cold, parental history of asthma, and residence (exposure) in the current home since 2 years of age or earlier. Information on potentially confounding exposures or characteristics included maternal smoking while pregnant with the child, current second-hand tobacco smoke exposure, family income and responding parent’s education, current coverage of the child by a health insurance plan, and housing characteristics, which included pets inside the home (dog, cat, bird, other furry or hairy pets, or other pets), cockroaches, rats or mice, carpeting, water damage or mold or mildew in the home since the child lived there, use of an air conditioner, second-hand tobacco smoke, and a combustion source for nitrogen dioxide in the home (a gas oven or stove or heating unit with a pilot light).

### Statistical analysis.

The odds ratio (OR) for each distance category was estimated with residences further than 300 m as the reference group, using logistic regression. All models were adjusted for the child’s age, sex, race, community, and language of questionnaire completion. To assess the effect of long-term and early-life exposure, some analyses were stratified into children living since 2 years of age or younger at the same residence and those moving to the current residence at a later age. Confounding was evaluated by assessing whether the coefficient of the log OR for exposure changed by > 10% after adding an additional covariate to this basic model. We assessed effect modification by parental history of asthma and the child’s history of allergic symptoms and sex by modeling the interaction of the potential effect modifier with exposure category (or with traffic-modeled exposure, as described in the online supplemental material) and by examining the effects of exposure by strata.

We also fitted logistic additive models (Hastie and Tibshirani 1990) to assess the functional relationship between childhood asthma and proximity to major roads. These models used the smoothing spline with 3 degrees of freedom for the continuous distance from major road and used the same adjustment variables as in the linear logistic models described above.

Significance was defined as two-sided *p* < 0.05 for all analyses. The logistic additive models were fitted using the S-plus programming language (Venables et al. 2002). All other analyses were performed using the Statistical Analysis System (SAS version 9.0; SAS Institute Inc., Cary, NC).

## Results

Of the 5,341 children completing a questionnaire and informed consent, 4,762 had an address that could be accurately matched and geocoded. Among these, there were 650 reports of ever physician-diagnosed asthma (14%); 577 cases of prevalent asthma (13%) based on current severe symptoms, use of controller medications, or lifetime asthma with current wheeze; and 682 children with current wheeze during the previous year (15%). Although there was some overlap of these phenotypes, 38% of children with lifetime asthma had no current wheeze, 16% with prevalent asthma had no current wheeze (based primarily on use of controller medications in prevalent asthma), and 17% of prevalent asthma cases had no lifetime reported doctor diagnosis of asthma. The mean (± SD) age was 6.5 ± 0.68 years. The frequency of other characteristics of children, parents, and households is shown in Table 1. Most children were Hispanic, and almost one-quarter of parents completed a questionnaire in Spanish. Eighteen percent of parents reported that annual household income was < $15,000, and 22% had less than a high school education. Forty-two percent of children had lived at the same address since 2 years of age or younger.

The mean (± SD) distance from the child’s residence to a major road was 418 ± 519 m (median, 254 m; range, 0.02–7,516 m). (Error in precisely locating homes and roadways accounted for distances less than the 13-m offset from the street used in geocoding residences.) Most residences (56.6%) were within 300 m of a major road: 25.2% were between 150 and 300 m, 16.4% between 75 and 150 m, and 15% within 75 m.

The risk of asthma-related outcomes was associated with residential distance to a major road (Table 2). Compared with those living at least 300 m from a major road, there were increased risks for all three outcomes among children within 75 m. For both prevalent asthma and current wheeze, there was increasing risk with decreasing residential distance to the roadway. Among long-term residents (living since 2 years of age at the same home), risk was increased only among those living within 75 m of a major road, and the ORs were slightly larger than the corresponding ORs in the entire population. Confounding by housing characteristics or other covariates from Table 1 was assessed among long-term residents, and the effect of living within 75 m of a major road was not substantially changed.

We examined interactions of exposure with the susceptibility factors in the sample restricted to long-term residents, because exposure in this group was more likely to have been accurately assigned for the period during which asthma developed than for children moving later. Parental asthma modified the effect of living within 75 m of a major road (Table 3). There were almost 2-fold (lifetime asthma) to almost 3-fold increased risks (current wheeze) associated with this exposure, but only among those children without a parental history of asthma. The interaction of parental history with residential proximity within 75 m was significant for prevalent asthma (1 degree of freedom, Wald chi-square 4.39; *p* = 0.04) and for current wheeze (*p* = 0.01), but not for lifetime asthma.

Among long-term residents who had no allergic symptoms, greater than 2-fold increased risks of all three outcomes were associated with living in a residence within 75 m of a major road (Table 4). However, there were no significant interactions of allergy with this exposure for any of the three outcomes.

Among boys, there was little evidence of increased risk associated with residential distance to a major road (Table 5). Among girls, strong associations with living within 75 m of a major road were observed for all three outcomes, and the difference between boys and girls was significant for lifetime asthma (1 degree of freedom interaction, *p* = 0.02).

**Figure 2:**
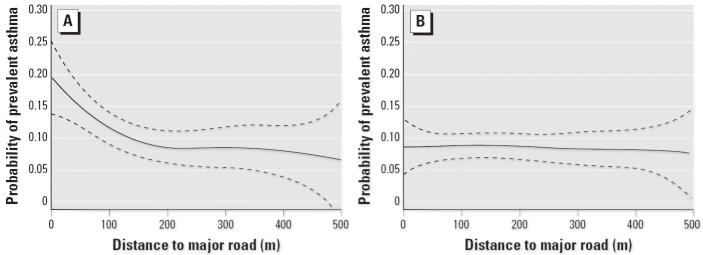
Prevalence of asthma by distance of residence to a major road within 500 m, among long-term (*A*) and short-term (*B*) residents with no family history of asthma. Dotted lines indicate 95% confidence interval.

Among children with no family history of asthma, we examined further the relationship of asthma and distance to a major road within 500 m of the home, using smoothed models. Among long-term residents, an increasing rate of prevalent asthma was observed with residential proximity to the nearest major road, and the risk decreased to background levels at 150–200 m (Figure 2). This trend was observed only among children living at the same address since 2 years of age. Children moving to the current residence after 2 years of age showed no effect of proximity to a major road. A similar pattern of effects was observed for lifetime asthma and wheeze (data not shown).

The effects of pollutants in fresh traffic exhaust modeled from traffic volume, distance, and meteorology were generally consistent with those observed for proximity to a major road (see online supplemental material). There were significant associations of nonfreeway (but not of freeway or total) traffic-modeled exposure with prevalent asthma and current wheeze, and these effects were stronger in long-term residents (Table S-2 in the online supplemental material). The stratum-specific pattern of traffic-modeled effects was also stronger in those with no parental history and with no allergic symptoms and among girls (Table S-3 in the online supplemental material).

## Discussion

Asthma and wheeze were strongly associated with residential proximity to a major road. These associations were strongest among children with no parental history of asthma who had lived at the same address since early in life. In this group, the highest risk occurred adjacent to the major road, and risk decreased to background rates at 150–200 m from the road. Larger risks of asthma associated with long-term residence within 75 m of a major road were observed among girls than among boys.

If traffic-related pollutants were responsible for the observed associations with asthma, the increased risk among the longer-term residents might be expected because they had a larger cumulative exposure to the pollutant indicators used in this analysis. However, the absence of any effect of a major road among children moving to their residence after 2 years of age (Figure 2) may indicate vulnerability during the prenatal period or infancy. Although the study design did not allow us to distinguish between these two possibilities, there is evidence that other early-life exposures may increase the risk of asthma (Martinez 1999). Recent case–control and cohort studies have found an increased risk of asthma with early-life exposure to local residential traffic-related pollutants (Brauer et al. 2002; Zmirou et al. 2004). In addition, several recent studies suggest that early-life (especially *in utero*) exposure to tobacco smoke, which like fresh vehicular exhaust is a complex mixture of air pollutants, is more strongly associated with increased risk of subsequent asthma than is exposure later in childhood (Gilliland et al. 2001, 2002). The larger effect of proximity to a major roadway among girls in our study also is consistent with previous reports (Oosterlee et al. 1996; Pershagen et al. 1995; Shima et al. 2003; van Vliet et al. 1997; Venn et al. 2001).

We previously found that children with an increased risk of incident asthma associated with exercise in high-ozone environments were less likely to have a parental history of asthma (McConnell et al. 2002), and another recent study found that the risk of traffic-associated prevalent asthma was larger in children without parental history (Gordian et al. 2005). However, both family history of asthma and child allergy are strong risk factors for asthma independent of exposure to air pollution (London et al. 2001; Peden 2000). In our study, among long-term residents living > 300 m from a major road, parental history was associated with a 3.6-fold increased risk of prevalent asthma [95% confidence interval (CI), 2.3–5.8] and child symptoms of allergy with a 6.4-fold increased risk (95% CI, 4.5–9.1). Therefore, one possible explanation for the larger effects of traffic exposure in children without these strong risk factors is that other risks, for example, dietary factors, indoor allergens, or other environmental exposures, produced asthma in the high-risk group, regardless of traffic-related exposures. It is possible that, among those with parental asthma or allergic symptoms, there was no additional risk of childhood asthma associated with traffic or that any small additional effect of traffic was undetectable in the high background rate of asthma in these children.

Parental history of asthma is an indication of genetic susceptibility, so the absence of risk among those with parental history may also indicate that asthma caused by pollutants in fresh traffic exhaust is less likely to be inherited, or at least is not mediated through the same genetic pathways that account for asthma in the parents of these children. Nonallergic asthma is one possible alternative pathway, which may be consistent with the stronger observed effect of traffic among children without hay fever or other allergic symptoms. Like parental history of asthma, allergic symptoms are associated with atopic asthma (Peden 2000). Atopy is characterized by a positive skin test or immunoglobulin E–specific response to environmental allergens. Recent studies indicate that nonallergic asthma (without airway eosinophilia or atopy) may account for as much as half of all asthma (Beasley et al. 2001; Douwes et al. 2002; Pearce et al. 1999), and it has been suggested that risk factors for this asthma phenotype, including particulate air pollution, may differ from those for allergic asthma (Douwes et al. 2002). Some studies of the risk of asthma and wheeze due to second-hand smoke, another mix of oxidant pollutants, have shown stronger effects among children without atopy or atopic symptoms (Kershaw 1987; Palmieri et al. 1990; Strachan and Cook 1998; Strachan et al. 1996a, 1996b). In addition, drug-induced and occupational asthma commonly occur in the absence of atopy, and many of the exposures responsible for “irritant-induced asthma” in the workplace are also present in the general population (Gautrin et al. 2003; Kitani et al. 1993). However, in other studies, stronger associations of asthma and wheeze with traffic-related pollutants were found among atopic children (Janssen et al. 2003; Zmirou et al. 2004) and with second-hand tobacco smoke exposure among children with an atopic parent (Jaakkola et al. 2001). In addition, laboratory evidence suggests that exposure to oxidant air pollution promotes the effect of allergens on asthma severity and on the pathogenesis of asthma (Jenkins et al. 1999; Kehrl et al. 1999; Li et al. 2003; Schelegle et al. 2003). Based on these studies, an effect of traffic-related pollutants might have been expected to be stronger among children with allergy. Further investigation is warranted to identify the reason for the apparent susceptibility of children without allergy and parental history of asthma in our study. Better phenotypic characterization of atopy both in the study children and in their parents and of allergen exposure in children would be useful to interpret the relationship of these characteristics to traffic and asthma.

In a previous cohort in the Children’s Health Study, we observed strong associations of lifetime asthma with residential ambient NO_2_, an indicator of variability within communities of traffic-related pollutants, which was measured at a sample of homes (Gauderman et al. 2005). Measured NO_2_ was moderately correlated with total traffic-modeled pollution (*R* = 0.59). Strong associations also were observed with residential distance to a freeway and with traffic-modeled exposure from freeways (but not from non-freeway traffic–modeled pollution). We have now extended these observations to a larger population and to residential distance to other major roadways. The association of asthma in our new cohort with non-freeway traffic–modeled exposure, but not with freeway-modeled exposure, may reflect differences in the distribution of freeways and major roads around homes in the different cohorts. The association of asthma with non-freeway traffic–modeled exposure is consistent with the observed association with distance to a major road, because there were few children within 75 m of a freeway in our study. Residential distance to a major roadway also is computationally easier to estimate from data that are more readily available than the meteorologic and traffic volume data required to model exposure. An increased risk associated with proximity to a major roadway also is more easily explained to policy makers and to the general public than is the risk associated with traffic-modeled exposure.

Our results are also consistent with several European studies that found increased risks of childhood asthma with increased traffic counts in close proximity to the home (Morris et al. 2000; Nicolai et al. 2003; van Vliet et al. 1997; Venn et al. 2001; Zmirou et al. 2004). One large British study that focused on traffic within 150 m of children’s homes found a gradient in risk that increased markedly with decreasing residential distance to a main road (Venn et al. 2001). There have been few other studies of traffic and childhood asthma in the United States. A recent study in northern California found an association between measured traffic-related pollutants at schools and childhood asthma (Kim et al. 2004). However, another large study in southern California based on records of children covered by Medicaid (public insurance for low-income persons) found no association between asthma prevalence and traffic counts within 168 m of the home, although an association with asthma medication was observed (English et al. 1999). Some of the inconsistencies in the literature could perhaps be explained by the failure of many studies to account for the pattern of effect modification by parental history of asthma and by age and duration of residential exposure to traffic-related pollutants that vary markedly at different locations. The larger effects of traffic in girls has been observed in previous studies of traffic and asthma and related symptoms, but the reason for the apparent susceptibility of girls is not known (Oosterlee et al. 1996; Pershagen et al. 1995; Shima et al. 2003; van Vliet et al. 1997; Venn et al. 2001).

A causal relationship between asthma and traffic-related exposures is biologically plausible, because ambient particulate matter and other oxidant pollutants have been shown to elicit responses relevant to the pathogenesis of asthma (Li et al. 2003). In addition, studies in southern California and elsewhere have shown that the largest gradients in traffic-related pollutants occur within the 150–200 m from roadways over which we saw decreasing risk of asthma (Gilbert et al. 2003; Zhu et al. 2002). In studies in which NO_2_ and other markers of traffic-related exposure have been measured in close proximity to major roadways, variability has usually been best explained by traffic volume within 300 m (Briggs et al. 2000; Gilbert et al. 2003; Ross et al. 2005), although weaker correlations have also been observed over longer distances from the highest volume traffic corridors (Gauderman et al. 2005; Gilbert et al. 2003, 2005; Ross et al. 2005).

We considered bias as an explanation for our results. Parents with asthma who were susceptible to environmental triggers might have selected homes away from major roads, perhaps even before the children were born. If the children of these parents had high rates of asthma, this might have explained the observed lack of effect of a major road in families with parental asthma. There is some evidence that parents may intervene to reduce household exposure to indoor allergens, another perceived risk for asthma and asthma severity (Almqvist et al. 2003; van Strien et al. 2002). However, this bias is unlikely to explain our results, because we examined and found no significant differences in rates of parental history of asthma by exposure to a major road (data not shown). Selection bias related to factors influencing participation could not be evaluated, because characteristics of nonparticipants are not known. However, there were some modest differences between participants whose addresses could be geocoded, who were of higher socioeconomic status than were participants whose addresses could not be geocoded. For example, of those with family income < $7,500, 85% could be geocoded, compared with 93% of those with ≥$100,000. Of those without insurance, 85% could be geocoded, compared with 90% of those with insurance. The differences between those whose homes could and could not be geocoded were heavily influenced by 299 subjects (of 579 total that could not be geocoded) who completed a questionnaire but did not provide an address. However, none of the three asthma outcomes was associated with absence of a home geocode, and the associations of asthma with traffic were robust to our adjustment for socioeconomic status. It has also been suggested that traffic-related noise might cause asthma through a pathway mediated by stress (Ising and Ising 2002). However, to date there is little evidence to support this hypothesis. Other potential confounders, including sociodemographic factors, second-hand or *in utero* tobacco smoke exposure, or housing characteristics that are commonly associated with asthma also did not explain our results. A final possible limitation to the interpretation of these results is the assessment of asthma by questionnaire. However, self-report of physician-diagnosed asthma has been reported to accurately reflect what physicians have told the patient, at least in adults, and validity of questionnaires as reported by repeatability of response is good (Ehrlich et al. 1995). For these reasons, self-report of physician-diagnosed asthma has been widely used in epidemiologic studies and has been recommended as the preferred outcome assessment for use in large population-based studies, because a more precise diagnosis is not available (Burr 1992). In addition, the consistency of associations with lifetime asthma, prevalent asthma based on a combination of symptoms reporting and doctor diagnosis, and recent wheezing suggests that diagnostic bias is unlikely to have explained the observed results.

We conclude that living in a residence with more nearby traffic increases the risk of childhood asthma. Children with no parental history of asthma who had long-term residential exposure (or early-life exposure) constituted a susceptible population, and the risk was larger for girls than for boys. Because a substantial number of southern California children live near a major road, this exposure is potentially an important public health problem that could be remediable by transportation and residential development policy and by more effective control of vehicular emissions. Among those long-term residents with no parental history of asthma who lived within 75 m of a major road, 59% of asthma was attributable to residential proximity to the road. Further investigation is warranted to understand why the absence of parental asthma history increased susceptibility to traffic-related exposure.

## Supplementary Material

Supplemental Figures and Tables

## Figures and Tables

**Table 1:** Demographic characteristics and potential confounders or susceptibility factors.

Characteristic	No. (%)^a^
Child	
Sex	
Male	2,425 (51)
Female	2,295 (49)
Race	
North American Indian	44 (0.93)
Asian	170 (3.6)
Black	197 (4.2)
Hispanic white	2,617 (55)
Non-Hispanic white	1,682 (35)
Other	32 (0.67)
Health insurance	3,985 (88)
Long-term residence	1,856 (42)
Allergy	1,834 (44)
*In utero* tobacco smoke	360 (7.9)
Parent	
Spanish questionnaire	1,091 (23)
Family income	
< $7,500	279 (6.9)
$7,500–14,999	456 (11)
$15,000–29,999	692 (17)
$30,000–49,999	709 (18)
$50,000–74,999	726 (18)
$75,000–99,999	535 (13)
≥$100,000	639 (16)
Parental education	
< 12th grade	982 (22)
Grade 12	880 (20)
Some post-high school	1,681 (38)
Four years of college	512 (11)
Some postgraduate	417 (9.3)
Parental asthma	965 (23)
Home	
Any pet	2,479 (54)
Dog	1,337 (29)
Cat	841 (18)
Bird	462 (10)
Cockroach	487 (11)
Mice	366 (8.1)
Rats	189 (4.2)
NO_2_ source	3,358 (72)
Air conditioner	2,763 (60)
Carpeting	4,230 (92)
Water damage	653 (14)
Mold or mildew	1,068 (25)
Second-hand smoke	794 (18)

a Total (% of total) with each characteristic; denominator varies due to missing values or “don’t know” responses.

**Table 2:** Association of asthma and wheeze with distance to a major road [OR (95% CI)].^a^

Major road distance (m)	No.^b^	Lifetime asthma	Prevalent asthma	Current wheeze
All participants				
> 300	2,058	1.00	1.00	1.00
150–300	1,193	0.92 (0.73–1.15)	1.04 (0.82–1.33)	1.02 (0.82–1.27)
75–150	778	1.06 (0.82–1.36)	1.33 (1.02–1.72)*	1.30 (1.02–1.66)*
< 75	713	1.29 (1.01–1.66)*	1.50 (1.16–1.95)**	1.40 (1.09–1.78)**
Long-term residents				
> 300	813	1.00	1.00	1.00
150–300	483	0.86 (0.59–1.24)	0.83 (0.56–1.21)	0.97 (0.69–1.38)
75–150	294	1.03 (0.68–1.56)	1.09 (0.71–1.66)	1.09 (0.73–1.62)
< 75	266	1.46 (0.98–2.17)	1.64 (1.10–2.44)*	1.67 (1.14–2.43)**

a Adjusted for age, sex, language of questionnaire, community, and race.

b Total exposed in each category of distance to a major road.

* *p* < 0.05;

** *p* < 0.01.

**Table 3:** Association of asthma and wheeze with distance to a major road among long-term residents, by parental history of asthma [OR (95% CI)].^a^

Major road distance	No parental asthma (*n* = 1,330)	Parental asthma (*n* = 380)
Lifetime asthma^b^		
> 300 m	1.00	1.00
150–300 m	1.06 (0.65–1.71)	0.62 (0.30–1.25)
75–150 m	1.13 (0.64–1.97)	0.75 (0.34–1.63)
< 75 m	1.85 (1.11–3.09)*	1.03 (0.47–2.24)
Prevalent asthma		
> 300 m	1.00	1.00
150–300 m	0.94 (0.57–1.58)	0.67 (0.33–1.37)
75–150 m	1.21 (0.69–2.14)	0.80 (0.37–1.74)
< 75 m	2.46 (1.48–4.09)**	0.79 (0.34–1.82)
Current wheeze		
> 300 m	1.00	1.00
150–300 m	1.02 (0.64–1.64)	0.96 (0.51–1.80)
75–150 m	1.37 (0.81–2.31)	0.88 (0.42–1.83)
< 75 m	2.74 (1.71–4.39)**	0.87 (0.40–1.90)

a Adjusted for age, sex, language of questionnaire, community, and race.

b Participants from Lake Arrowhead were excluded from the model for stratum with no parental asthma, because otherwise the model failed to converge.

* *p* < 0.05;

** *p* < 0.01.

**Table 4:** Association of asthma and wheeze with distance to a major road among long term residents, by child’s history of allergy [OR (95% CI)].^a^

Major road distance (m)	No allergic symptoms (*n* = 942)	Allergic symptoms (*n* = 723)
Lifetime asthma^b^		
> 300	1.00	1.00
150–300	0.92 (0.43–1.97)	0.87 (0.53–1.41)
75–150	1.04 (0.41–2.62)	0.96 (0.57–1.61)
< 75	2.27 (1.04–4.94)*	1.31 (0.76–2.25)
Prevalent asthma		
> 300	1.00	1.00
150–300	0.98 (0.42–2.26)	0.77 (0.46–1.27)
75–150	0.81 (0.25–2.55)	1.01 (0.60–1.69)
< 75	2.52 (1.07–5.93)*	1.29 (0.76–2.21)
Current wheeze		
> 300	1.00	1.00
150–300	1.50 (0.72–3.12)	0.80 (0.50–1.28)
75–150	0.72 (0.23–2.25)	1.03 (0.63–1.68)
< 75	2.58 (1.14–5.86)*	1.25 (0.75–2.07)

a Adjusted for age, sex, language of questionnaire, community, and race.

b Participants from Lake Arrowhead were excluded from models for stratum without allergy for prevalent and lifetime asthma, because otherwise the models failed to converge.

* *p* < 0.05.

**Table 5:** Association of asthma and wheeze with distance to a major road among long term residents, by child’s sex [OR (95% CI)].^a^

Major road distance (m)	Boys (*n* = 945)	Girls (*n* = 901)
Lifetime asthma^b^		
> 300	1.00	1.00
150–300	0.87 (0.54–1.40)	0.88 (0.48–1.61)
75–150	1.15 (0.69–1.92)	0.68 (0.31–1.48)
< 75	0.94 (0.54–1.64)	2.51 (1.39–4.54)**
Prevalent asthma		
> 300	1.00	1.00
150–300	0.77 (0.46–1.30)	0.90 (0.50–1.61)
75–150	1.37 (0.82–2.31)	0.53 (0.23–1.24)
< 75	1.31 (0.75–2.29)	2.13 (1.18–3.85)*
Current wheeze		
> 300	1.00	1.00
150–300	0.96 (0.60–1.53)	0.99 (0.58–1.69)
75–150	1.27 (0.77–2.10)	0.72 (0.35–1.46)
< 75	1.41 (0.84–2.37)	1.95 (1.11–3.41)*

a Adjusted for age, language of questionnaire, community, and race.

b Participants from Lake Arrowhead were excluded from model for stratum with girls for lifetime asthma, because otherwise the model failed to converge.

* *p* < 0.05;

** *p* < 0.01.
