# Supplementary material for: Traffic, Susceptibility, and Childhood Asthma
Source: Environ Health Perspect. 2006 Feb 16;114(5):766–72. doi: 10.1289/ehp.8594 (PMC1459934; doi:10.1289/ehp.8594)
Supplement: Supplemental Figures and Tables [file ehp0114-000766s1.pdf]

Online Supplement

Assessment of Modeled Exposure to Traffic-related Pollutants

There are many alternative indicators that have been developed to assess local exposure to fresh traffic-related pollutants (Jerrett et al. 2005). Distance to a major roadway was selected because it was found to be associated with asthma in previous studies and because it is an index that could more easily be estimated accurately in other locations than more complex models. We also examined the effect of modeled estimates of levels of exposure that accounted for traffic volume and meteorology to see if there was a consistent pattern of associations to those observed for the simpler metric. Briefly, homes were geo-located, as described for the estimation of residential distance to a major road. Annual average daily traffic volumes were obtained from the California Department of Transportation Highway Performance Monitoring System (CalTrans 2002). The year 1997 was selected to represent early life exposure in this cohort, but in the established communities in southern California where this study was conducted there was relatively little change from 1997 over the lifetime of these children prior to study enrollment. Using previously described methods, the traffic volumes were transferred from the Department of Transportation roadway network to the Tele Atlas networks (Wu et al. 2005). Residential exposure to local traffic-related pollutants was estimated from dispersion models that incorporate distance to roadways, vehicle counts, vehicle emission rates, and meteorological conditions (Benson 1989). We estimated separately the contribution of local traffic on freeways and on all other roadways to concentrations of several pollutants, including carbon monoxide, nitrogen dioxide, total oxides of nitrogen, elemental and organic carbon and particulate matter with aerodynamic diameter less than 10 and less than 2.5 $\mu g/m^3$**.** These estimated pollutant exposures should be regarded as indicators of annual average incremental increases due to primary emissions from local vehicular traffic on top of background ambient levels. Modeled NO_x, for example, which we use to evaluate associations with asthma, represented only the effect of the incremental contribution of local traffic to a more homogeneous community background

concentration of NO_x that included both primary and secondary pollution resulting from long range transport and regional atmospheric photochemistry. All the various traffic-modeled pollutants were highly correlated (R>0.90). Therefore, modeled NO_x represented primary local NO_x from vehicular traffic, these other highly correlated pollutants in fresh traffic exhaust, and probably other pollutants for which we did not estimate exposures. Because it is not possible to distinguish the effects of these different pollutants in fresh traffic exhaust, we refer to this exposure metric as local traffic-modeled pollution, rather than as a specific pollutant. We have previously found moderate correlation (R=0.59) of these modeled estimates with variation within communities in measured NO_2, an indicator of traffic-related pollutants (Gauderman et al. 2005).

The odds ratio for each traffic-modeled pollution metric (freeway, non-freeway and total) was estimated using logistic regression and scaled to the interquartile range for the total traffic-modeled pollution. Interaction was assessed for each traffic-modeled metric with each characteristic of susceptibility. Estimates specific to each dichotomous marker for susceptibility (for example parental history) were fit in a single model with adjustment for confounders and basic design variables.

Results

The traffic-modeled pollution contribution from freeways was generally greater and was more markedly skewed to the right than the contribution from non-freeway traffic (Table S-1). These metrics were not strongly correlated with distance to a major road (Pearson R=-0.28 for freeway-modeled pollutants and -0.40 for non-freeway pollutants) or with each other (R=0.31). Total traffic-modeled pollution was very strongly correlated with freeway pollution (R=0.94) and strongly correlated with non-freeway pollution (R=0.62).

There were significant associations between the contribution of non-freeway traffic-modeled exposure and prevalent asthma and current wheeze, which were significant (Table S-2). Among long term residents**,** the effect estimates were modestly stronger for

all three outcomes. There was no association of asthma with the freeway contribution to traffic-modeled exposure.

Among long term residents the effect of non-freeway traffic was generally larger among those with no parental history of asthma, and the interaction was significant for lifetime asthma (Table S-3; interaction P = 0.03). There was no evidence of effect modification by parental history for the freeway or total traffic-modeled exposure (data not shown). There were larger effects of non-freeway traffic-modeled exposure among those without allergic symptoms than among those with allergic symptoms, but there were no significant interactions. For freeway traffic-modeled exposure, however, the odds ratios for current wheeze were 0.87 (95% C.I. 0.57,1.32) and 1.40 (95% C.I. 0.98, 2.01), among those with and without allergic symptoms, respectively (interaction P = 0.04). There were generally larger effects of non-freeway modeled exposure in girls, but there were no significant interactions of gender with any traffic-modeled metric.

REFERENCES

Benson P. 1989. Caline4 -- A dispersion model for predicting air pollution concentration near roadways. Sacramento, California: California Department of Transportation, Office of Transportation Laboratory.

CalTrans (California Department of Transportation). 2002. California Motor Vehicle Stock, Travel and Fuel Forecast. Available: http://www.dot.ca.gov/hq/tsip/otfa/mtab/MVSTAFF/MVSTAFF02.pdf [accessed 13 February 2006].

Gauderman WJ, Avol E, Lurmann F, Kunzli N, Gilliland F, Peters J, et al. 2005. Childhood Asthma and Exposure to Traffic and Nitrogen Dioxide. Epidemiology 16(6):737-743.

Jerrett M, Arain A, Kanaroglou P, Beckerman B, Potoglou D, Sahsuvaroglu T, et al. 2005. A review and evaluation of intraurban air pollution exposure models. J Expo Anal Environ Epidemiol 15(2):185-204.

Wu J, Funk T, Lurmann F, Winer A. 2005. Improving spatial accuracy of roadway networks and geocoded addresses. Transactions in GIS 9(4):585-601.

Table S-1: Distribution of traffic-modeled exposure to freeway and non-freeway traffic sources

Traffic type[a]	Mean	Median	Interquartile Range	Minimum, Maximum
Freeway	15.1	8.9	19.2	(0,223)
Non-freeway	10.7	9.1	11.6	(0.11,81)
Total	25.9	19.8	28.7	(0.35,238)

[a]In ppb for modeled NO_x

Table S-2: Association of asthma and wheeze with traffic modeled pollution

	Lifetime Asthma		Prevalent Asthma			Current Wheeze		
	O.R.[a]	(95% C.I.)	O.R.[a]	(95% C.I.)		O.R.[a]	(95% C.I.)	
**All Participants**								
Traffic modeled exposure								
Freeway modeled pollution	1.07	(0.90 , 1.28)	1.00	(0.83 , 1.22)		1.04	(0.87 , 1.24)	
Non-freeway modeled pollution	1.21	(0.84 , 1.75)	1.64	(1.14 , 2.36)	**	1.67	(1.18 , 2.34)	**
Total traffic modeled pollution	1.09	(0.94 , 1.28)	1.10	(0.94 , 1.30)		1.14	(0.98 , 1.32)	
**Long Term Residents**								
	Lifetime Asthma		Prevalent Asthma			Current Wheeze		
	O.R.[a]	(95% C.I.)	O.R.[a]	(95% C.I.)		O.R.[a]	(95% C.I.)	
Freeway modeled pollution	0.91	(0.67 , 1.23)	0.98	(0.72 , 1.33)		1.04	(0.78 , 1.38)	
Non-freeway modeled pollution	1.34	(0.70 , 2.53)	2.07	(1.12 , 3.83)	*	1.79	(0.99 , 3.25)	
Total traffic modeled pollution	0.97	(0.75 , 1.26)	1.11	(0.86 , 1.43)		1.14	(0.89 , 1.45)	

[a]O.R. (95% C.I.) odds ratio (95% confidence interval), adjusted for age, sex, language of questionnaire, community, and race; traffic modeled local contribution to exposure scaled over interquartile range for total traffic-modeled pollution
*p<.05; **p<.01

Table S-3: Association of asthma and wheeze with non-freeway traffic-modeled pollution by susceptibility factors among long term residents

	Lifetime Asthma		Prevalent Asthma		Current Wheeze	
	O.R.[a]	(95% C.I.)	O.R.[a]	(95% C.I.)	O.R.[a]	(95% C.I.)
No parent history[b]	2.16	(1.04 , 4.47) *	2.80	(1.38 , 5.72) **	2.09	(1.04 , 4.20) *
Parent history	0.63	(0.22 , 1.82)	1.21	(0.46 , 3.20)	1.63	(0.67 , 3.97)
No allergy[b]	1.94	(0.71 , 5.29)	3.27	(1.25 , 8.50) *	2.67	(1.07 , 6.62) *
Allergy	1.30	(0.57 , 2.98)	2.10	(0.93 , 4.74)	1.55	(0.70 , 3.41)
Boys	1.25	(0.5 , 3)	1.30	(0.53 , 3.15)	1.20	(0.52 , 2.80)
Girls	1.74	(0.7 , 4.6)	2.98	(1.13 , 7.88) *	2.16	(0.84 , 5.60)

[a]O.R. (95% C.I.) odds ratio (95% confidence interval) for non-freeway traffic modeled local contribution to exposure scaled over interquartile range for total traffic-modeled pollution, adjusted for age, sex, language of questionnaire, community, and race

[b]Participants from Lake Arrowhead were excluded from models for stratum without family history for lifetime asthma and from models without allergy for current wheeze, because otherwise the models failed to converge.

*p<.05; **p<.01
